# Observations on Incentives to Improve Population Health

**Published:** 2010-08-15

**Authors:** J. Michael McGinnis

**Affiliations:** Institute of Medicine

## Initial Reflections

The pace of progress in population health can be influenced by the incentives in play and the metrics that trigger them. The MATCH (Mobilizing Action Toward Community Health) articles in this issue of *Preventing Chronic Disease* explore the use of incentives to improve population health and hold implications for the development and application of the measures to which they are linked. Metrics in population health can serve to draw and focus attention, encourage action, and direct rewards and penalties. When those rewards and penalties take on an economic dimension, the results can be powerful.

This potential application of population health measures is especially important if the aim is to transform the allocation of social energy and resources, as it clearly must be. Currently, our national health investment profile is deeply flawed — more than 95% of every health dollar goes to treatment rather than prevention. In a system in which all our salient incentives are structured to reward volume over value, we miss virtually no opportunity to treat disease, often unsuccessfully or erroneously.

On the other hand, each day we miss countless opportunities to prevent disease and promote health. If we seek to reform health care payment systems to yield better health returns, investment in prevention has to move to the highest — not lowest — priority. If our aim is to fashion the health equivalent of indicators that shape our economic policies, the most rational social investment strategy would center around prevention and our health care payment system would follow suit.

A reformed health care payment system can advance health as the fundamental priority in 3 ways. First, every American should receive coverage for the clinical preventive services that are appropriate to him or her without copayment. Second, grant support should be set aside for community-based initiatives that are necessary to improve the health and health care of the community's residents. Finally, resources to address the overall health care needs of a population should be shaped by a blend of the community's health needs and efforts, as reflected by metrics that indicate trends for determinants of the population's health status.

## The Articles

The articles in this issue present a number of perspectives relevant to considering how incentives might work for population health improvement. Described below are common elements and how we might think about using incentives.

Haveman introduces the economist's perspective of the concept, structure, and function of incentives — financial and nonfinancial — including examples from education, jobs, and health (1). Mullahy reviews the conceptual challenges in transferring insights from targeting incentives for personal health services to possible effects on population health, including issues related to accounting for the production function for population health and the roles of multiple sectors (2). Rothschild shows the relevance of social marketing as a factor in improving population health (3).

Witte looks at performance metrics and rewards in education as a reference point for population health (4). Baxter identifies incentive options if no new resources are available, for example, using existing but unenforced requirements (such as those related to the nutritional content of school meals), using the purchasing power of government or emphasizing "cobenefits" (such as taxes on tobacco that offer disincentives and raise revenues) (5). Asch assesses the applicability of paying for performance in health care to population health (6).

Fox looks at the nature and evolving results of "triple aim" efforts, with emphasis on health care, population health, and cost reduction, including how a "value dividend" might most effectively be characterized (7). Oliver describes the potential incentives inherent in population health rankings such as MATCH, including how to link them to key uses such as identifying problems, setting agendas, and changing community policies (8). Smith reviews the European experience with setting heath targets, noting, for example, the challenges in setting the targets (which ones, outcomes vs process, how to quantify, cross-sector responsibilities) and in translating some of the key population health aims to the local level (9).

Each of the articles is rich with examples of economic incentives, such as  the use of graduate medical education payments by Medicare to teaching hospitals (1). Many of the examples, however, can have unintended consequences:

- The intent of developing the diagnosis-related groups (DRGs) — paying a flat fee for a group of services for a given condition — is to blunt the tendency of fee-for-service to increase service volume. Some "gaming" occurs, however, such as listing healthy patients under a more expensive DRG category or dividing the treatment into multiple admissions or episodes (6).
- Merit pay in education in Wisconsin did not appear to yield the educational value anticipated for teacher performance, judged by the year-to-year identification of high-performing teachers (4).
- The Child Nutrition Act has provisions for nutrition and wellness programs, but these are often unenforced because states view them as unfunded mandates. The situation is similar in the persistent number of eligible-but-unenrolled children in the Early Periodic Screening, Diagnosis, and Treatment Program and the State Children's Health Insurance Program (5).
- Pay-for-performance as a motivating strategy to improve clinical care may have perverse consequences. For example, providing extra payment based on the percentage of diabetes patients whose glycated hemoglobin levels are below 7% has led to clinician avoidance of difficult-to-manage patients and to overdiagnosing and overtreating patients with borderline levels (6).
- Prominent public reporting of coronary bypass graft death rates in New York State led to an increased number of operations in New York on patients with less severe illness and, alternatively, to referral of patients with more severe illness to border states for treatment (6).
- In assessing health system performance in the United Kingdom, where resources were allocated to perceived need, some managers disregarded the threat of damage to their reputations and were happy to use poor performance scores on what they viewed as unimportant processes as a strategy to get more resources, while other managers worked efficiently and received no reward for their superior performance (9).

## Common Elements in Considering Incentives

While the authors of these MATCH articles approached their assignments differently, they touch on common elements that should be considered in assessing the intended impact of incentives:

1. **Nature of the targeted actor.** Is the focus on a person making a personal decision, or is it an institutional decision maker or geographic collective? What is the relevant sector of action — health, education, environment, transportation?
2. **Nature of the targeted change.** Is change anticipated at a single locus (such as institutional, geographic, or cultural) or, as is more frequently the case, is it multilevel in nature?
3. **Choice of measures.** What measures will be used? Are they individual or are they summary in nature? What are the implications for their interpretation?
4. **Types of incentives.** Which of the multiple incentive approaches — financial, regulatory, legal, reputational, and educational — is most appropriate? Will the incentive be a reward or a penalty?
5. **Processes used.** Will recipients of the incentives participate in developing the incentive scheme, or will it be imposed with minimal consultation? Does the contemplated action directly target the desired outcome, or is it indirect — for example, clean indoor air laws to reduce tobacco use or revenue-enhancing excise taxes to reduce soda use?
6. **Decisional environment.** How supportive is the operative culture to direct or indirect social intervention? For example, how receptive will political and social leaders be to the health sector's seeking change in education, housing, or other social services, according to the potential effect on health?
7. **Funding stream involved.** Is the funding or support stream for the incentive likely to be episodic or sustained? Is it the product of a temporary public-private initiative? Is it an ongoing grant program? Is it embedded as part of a broad entitlement change?
8. **Possible unintended consequences.** What are the ways in which the contemplated incentive might distort the result or lead to new problems?

## Hierarchy of Potential Uses

Incentives, explicit or implicit, are inherent in metrics. Even independent of economic components, the mere establishment and monitoring of targets can impact reputation, recognition, and the inclination or disinclination toward alliances and can alter behavior. Because consequences, intended and unintended, can be both real and severe, care is needed in the choice of incentives. In effect, a certain hierarchy of consideration should be operative in their choice:

1. **Do no harm.** The golden rule of any policy is to ensure that its net result is salutary. Attention must first be devoted to understanding and assessing potential detrimental consequences, including consequences of inaccuracy and misuse, and taking steps to avoid them.
2. **Educate.** Choose measurement targets that can educate about issues. Some targets can make a difference in progress merely by being included in the metrics set.
3. **Signal.** Choose metrics that signal the importance of issues, through the structure and reporting of the effort.
4. **Celebrate.** Choose metrics that identify and celebrate the successes of prevention, when prevention's successes may be otherwise silent.
5. **Enable.** Choose metrics that can help forge partnerships and common bonds across sectors with mutual interests, for example, health with environment, education, and housing.
6. **Motivate.** Identify measures that can help motivate communitywide public action, through information that offers broad perspective about community opportunities and shortfalls, such as MATCH's potential provision of comparative population health information and community ranking.
7. **Empower.** Marshal community support to engage and act on issues with particular "public good" qualitities, such as advocacy for healthy school environments, clean water, clean air, and food safety.
8. **Reward.** Structure economic reward systems carefully, given the potential for distortion.
9. **Punish.** Shape sanctions or penalties when necessary, again carefully, given the potential for distortion.

This hierarchy of uses varies by circumstance. For example, punishment could be higher on the list in the case of egregious potential public threat, for example, the potential release of a populationwide health contaminant. Nonetheless, the hierarchy frames important starting considerations.

## Conclusion

Our understanding of how metrics and their incentives can enlighten, motivate, change, and advance population health will continue to mature. Addressing the challenges elucidated in the MATCH articles in this issue of *Preventing Chronic Disease* could refocus the resources available in the United States to improve population health.
